# Preparation of MnO_x_/CC Electrode by One-Step Electrodeposition for Electrochemical Detection of Cd^2+^ in Water

**DOI:** 10.3390/s25051415

**Published:** 2025-02-26

**Authors:** Jun Yin, Haiyang Huang, Cong Zhao, Haoyu Zhu, Hui Suo, Dong He, Chun Zhao

**Affiliations:** 1 State Key Laboratory of Integrated Optoelectronics, JLU Region, College of Electronic Science and Engineering, Jilin University, Changchun 130012, China; yinjun23@mails.jlu.edu.cn (J.Y.);; 2 Jilin Province Product Quality Supervision and Inspection Institute, No.2699, YiJu Road, Changchun 130103, China

**Keywords:** electrochemical sensor, cadmium ions, valence cycle of Mn, MnO_x_/CC

## Abstract

Transition metal oxides (e.g., MnO_x_) can effectively promote the redox reactions of heavy metal ions through abundant valence changes. However, relatively few studies have been conducted on the application of MnO_x_ for the detection of Cd^2+^ without pre-enrichment conditions. For this reason, in this study, MnO_x_ was grown in situ on a carbon cloth substrate by one-step electrodeposition. The effect of the valence composition of MnO_x_ and its variation on the Cd^2+^ without pre-enrichment detection performance was systematically investigated. The morphology, structure, and chemical composition of the materials were fully characterized by scanning electron microscopy (SEM), X-ray diffraction (XRD), and X-ray photoelectron spectroscopy (XPS). The results show that the deposition of MnO_x_ not only significantly increased the active surface area of the electrodes but also facilitated electron transfer through the valence transition of Mn^2+^/Mn^3+^↔Mn^3+^/Mn^4+^. The detection of Cd^2+^ in water samples can be successfully achieved without pre-enrichment, and the electrode has good stability and reproducibility. This study provides a new design idea for applying MnO_x_ electrodes in Cd^2+^ detection without pre-enrichment and provides a reference for further optimization of electrochemical sensors.

## 1. Introduction

Heavy metal pollution has become a major problem affecting the global environment and public health [1]. Cadmium is a typical heavy metal pollutant with high toxicity, bioaccumulation, and persistence [2,3]. Water is the main carrier of heavy metal ion enrichment; therefore, timely and accurate monitoring of the concentration of heavy metal pollutants in water is the first step in preventing and responding to heavy metal pollution. This is essential for controlling environmental pollution and protecting public health.

Traditional methods for detecting heavy metal ions, such as spectrometry, chromatography, and mass spectrometry, are known for their high sensitivity, good selectivity, and stability [4]. However, these techniques often require high-precision instrumentation and complex sample preparation processes [5], which require specialized personnel to operate. These characteristics limit the application of the above methods in rapid field testing. Electrochemical techniques are now a vital research area for the rapid detection of heavy metal ions, thanks to their simplicity, quick response times, low cost, and portability [6]. In electrochemical analysis, dissolution voltammetry is an analytical technique with high sensitivity [7,8]. Its detection process usually consists of two steps: pre-enrichment and dissolution. In the pre-enrichment stage, a fixed negative potential is applied to the solution, which causes the metal ions in the solution to be reduced and deposited to the metallic state on the electrode surface [9,10]. In this process, the metal ions are concentrated on the electrode surface, thereby substantially increasing the detection sensitivity. The pre-enrichment process usually takes time in the range of 120~600 s [11,12,13,14], reducing the detection efficiency. For example, Tang et al. [12] achieved simultaneous detection of Cd^2+^ and Pb^2+^ in water using PCys/Pβ-CD/GCE electrode at −1 V potential for 300s. If the cancellation of the pre-enrichment process is adopted, the number and time of electrode work can be reduced to increase the service life of the electrode. Therefore, it is important to study how to efficiently modify the electrode to promote the direct accumulation of heavy metal ions on the electrode surface [15], improve efficiency, and prolong the service life of the electrode, which is crucial for the rapid detection of heavy metal ions.

Transition metal oxides are regarded as effective materials for removing toxic heavy metal ions from groundwater that have high total dissolved solids [16]. The excellent performance of TMOs arises from the synergistic interaction between their electronic properties and surface morphology, which together influence the performance of the materials in terms of adsorption capacity, selectivity, and catalytic efficiency [17]. Additionally, TMOs have shown promising applications in electrochemical detection without needing pre-enrichment. Gao et al. [15] demonstrated that molybdenum-doped tungsten oxide enabled highly sensitive detection of heavy metal ions without the need for pre-enrichment. On the one hand, metal ions of transition metal oxides can participate in the redox reactions of target ions through valence changes. This property significantly improves the electron transfer efficiency of the electrode. For example, Li et al. [18] found that Ni doping promoted the valence transition between Co^2+^/Co^3+^ and improved the catalytic activity of Co_3_O_4_ nanosheets for the reduction of Hg^2+^ to Hg^0^. On the other hand, the multiple morphology and surface properties of TMOs (e.g., nanopore structure and defect sites) also play a crucial role in the selective adsorption of heavy metal ions [19]. Gao et al. [20] showed that oxygen vacancies can act as the main active sites of Hg^2+^. Redox reactions between Co^2+^/Co^3+^ and redox reactions between V^3+^/V^4+^ have a synergistic effect in the detection of Hg^2+^. Based on the above properties, TMOs provide an effective strategy for the development of highly sensitive and selective methods for the detection of heavy metal ions without pre-enrichment.

Manganese oxide (MnO_x_), a typical transition metal oxide, is widely utilized in various applications, including catalysis [21,22], battery materials [23,24], supercapacitors [25,26], and electrochemical sensors [27,28,29]. MnO_x_ also has the advantages of low cost, environmental friendliness, and high stability. Notably, the unique ability of MnO_x_ to undergo valence conversion among Mn^2+^, Mn^3+^, and Mn^4+^ allows for exceptional electron transfer efficiency in electrochemical reactions [30]. This characteristic significantly enhances its potential for electrochemical applications. In addition, by modulating the morphology of MnO_x_ (e.g., flake, wire, flower, sphere, etc.), the specific surface area and the number of active sites can be markedly increased, thus optimizing electrochemical performance. For example, MnO_2-x_ nanoflowers prepared by Du et al. [31] significantly enhance the cycling stability of supercapacitors by increasing the active area. Notably, it has been shown that MnO_x_ can also be applied as an adsorbent for wastewater treatment, exhibiting excellent adsorption performance for heavy metal ions such as Cu^2+^, Pb^2+^, Zn^2+^, Cd^2+^, and As^3+^ [32,33,34]. By rationally designing the morphology and valence ratio of MnO_x_ to enhance its sensitivity and detection efficiency in electrochemical sensors, new strategies and ideas are provided for developing high-performance sensors.

This study focuses on investigating the ability of MnO_x_ to detect Cd^2+^ without applying negative potential pre-enrichment of Cd^2+^ (known as without pre-enrichment). We used a one-step electrodeposition method to grow manganese oxide in situ on a carbon cloth substrate. MnO_x_/CC electrodes with different morphologies and valence compositions were prepared using different electrodeposition methods. The effects of morphology and valence on electrode performance were compared. The experimental results show that the MnO_x_−1 containing mixed valence states of Mn^2+^, Mn^3+^, and Mn^4+^ prepared using cyclic voltammetry has better detection performance. The electrode has a sea urchin-like morphology. The analysis showed that the valence change between Mn^2+^/Mn^3+^↔Mn^3+^/Mn^4+^ facilitates electron transfer, which in turn significantly enhances the redox reaction of Cd^2+^ and improves the electrode’s sensitivity. The electrode’s excellent reproducibility and stability indicate its potential for broad applications in detecting Cd^2+^.

## 2. Materials and Methods

### 2.1. Chemical Reagent

Manganese sulfate (MnSO_4_·H_2_O) was purchased from Beijing Chemical Factory. Anhydrous sodium sulfate (Na_2_SO_4_, 99%) was purchased from Tianjin Yongda Chemical Reagent Co. (Tianjin, China). Toluene (C_6_H_5_CH_3_) and acetone (CH_3_COCH_3_) were purchased from Xilong Scientific Co., Ltd. (Foshan, China). Glacial acetic acid, sodium acetate trihydrate (CH_3_COONa·3H_2_O), and potassium hydrogen phosphate trihydrate (K_2_HPO_4_·3H_2_O), along with potassium dihydrogen phosphate (KH_2_PO_4_), were obtained from Beijing Chemical Works Co. Ltd. (Beijing, China). Carbon cloth (W0S1011) with a relative hydrophilicity of 20 × 40 cm was purchased from Shengeruo Energy Mall (Suzhou, China). All the chemicals were used without further purification, and all aqueous solutions were prepared from deionized water.

### 2.2. Synthesis of MnO_x_/CC

Carbon cloths (1.5 cm × 2 cm) were first cleaned ultrasonically in a sequential process using toluene, acetone, ethanol, and deionized water, followed by drying at 60 °C. The carbon cloths were subjected to acid treatment by soaking them in a 3:1 (*v*/*v*) blend of concentrated nitric and sulfuric acids for 24 h following the drying process. Upon completion of the treatment, the carbon cloths were rinsed with deionized water until neutral. They were then stored in deionized water for future use.

MnO_x_-modified carbon cloth electrode (MnO_x_/CC) was prepared using the electrochemical method. A three-electrode configuration was used, with the working electrode being a carbon cloth (CC), the auxiliary electrode being a platinum sheet (1.5 cm × 1.5 cm), and the reference electrode being an Ag/AgCl electrode. All experiments were conducted in an EC Analyser electrochemical workstation. The electrolyte was created by dissolving 0.005 M manganese sulfate and 0.1 M sodium sulfate in 50 mL of deionized water while stirring thoroughly. The MnO_x_−1/CC electrode was prepared through four cycles of electrodeposition at a scanning rate of 50 mV/s within the potential range of −0.8 V to 1.4 V. To investigate the effects of diverse electrochemical preparation methods on the morphology and properties of MnO_x_, another series of experiments was conducted in the same electrolyte, involving electrodeposition at a potential of 1.4 V to obtain the MnO_x_−2/CC electrode. By comparing the effects of these two distinct electrodeposition methods, their influence on the growth morphology of MnO_x_ and its electrochemical properties was analyzed. Following electrodeposition, the electrodes were thoroughly rinsed with ethanol and deionized water and then dried at 60 °C to remove any residual moisture.

### 2.3. Electrochemical Testing

Electrochemical measurements were conducted using a three-electrode setup on a CHI660D electrochemical workstation (Shanghai Chenhua Instrument Co., Ltd., Shanghai China). The MnO_x_/CC electrode, with a working area of 0.5 × 0.5 cm^2^, acted as the working electrode. In contrast, a saturated calomel electrode (SCE) and a platinum sheet (1.5 × 1.5 cm^2^) served as the reference and counter electrodes, respectively. Differential pulse voltammetry was applied under optimized conditions to detect Cd^2+^ in aqueous samples.

### 2.4. Adsorption Experiment

The MnO_x_/CC electrode was immersed in an ABS buffer solution at pH 5.5, containing 5 μM Cd^2+^ under unapplied potential conditions, with stirring for 60 s. At the end of the reaction, the electrode was removed and dried at 60 °C, followed by X-ray photoelectron spectroscopy (XPS) analysis.

## 3. Results and Discussion

### 3.1. Structural and Morphological Features

**Figure 1:**
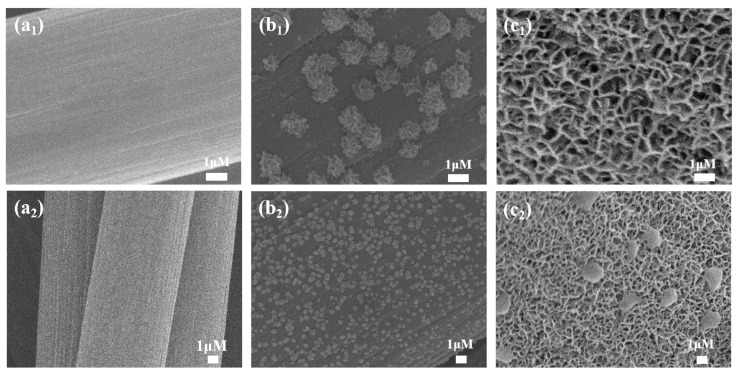
(**a_1_**,**a_2_**) SEM images of bare carbon cloth; (**b_1_**,**b_2_**) SEM image of MnO_x_−1/CC; (**c_1_**,**c_2_**) SEM images of MnO_x_−2/CC.

The present study investigates the effects of different electrodeposition methods on the morphology of MnO_x_/CC electrodes and their electrochemical properties. To this end, MnO_x_/CC electrodes were prepared using various electrodeposition means. Firstly, the microstructure of each electrode was precisely characterized using scanning electron microscopy (SEM). As illustrated in Figure 1(a_1_,a_2_), the bare carbon cloth electrode (CC) displays a characteristic carbon fiber braided structure featuring a smooth, uncontaminated carbon fiber surface with a well-defined structure. The MnO_x_−1/CC electrodes prepared by cyclic voltammetry show unique morphological features in Figure 1(b_1_,b_2_), where the surface of the carbon fibers is uniformly covered with sea urchin-like nanostructures. Cyclic voltammetry may keep the redox reaction of MnO_x_−1 going through periodic potential changes. The cyclic potential changes may alter the nucleation rate and crystal morphology, resulting in MnO_x_ particles that tend to agglomerate and form rough sea urchin-like morphology during the growth process. The surface of these sea urchin-like nanostructures is filled with rough bumps and depressions. This feature substantially enhances the electrodes’ specific surface area and offers additional active sites for heavy metal ion adsorption. The microstructures of the MnO_x_−2/CC electrodes prepared by constant-potential electrodeposition show irregular interconnected mesh with a small amount of lamellar structure, as shown in Figure 1(c_1_,c_2_), and their surface morphologies are significantly different from those of the electrodes prepared by cyclic voltammetry. It may be because the stable potential favors the continuous growth of MnO_x_−2 on the carbon cloth surface. The direction and rate of crystal growth are relatively more consistent, and it is easy to form a constant reticular structure. During the growth process, it may lead to some larger sheet stacking in the reticular structure due to the uneven local current density or the fluctuation of the ion concentration in the solution. This structural difference may have an important effect on the electrochemical performance of the electrodes.

**Figure 2:**
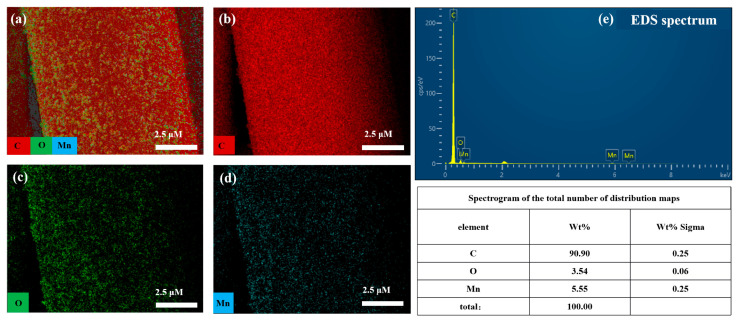
Elemental mapping of (**a**) MnO_x_−1/CC, (**b**) C, (**c**) O, (**d**) Mn, (**e**) EDS spectrogram.

To further verify the composition and elemental distribution of the MnO_x_−1/CC electrode, the electrode surface was characterized by energy spectrum analysis (EDS). Figure 2e shows the EDS spectra of MnO_x_−1/CC, which shows the major characteristic peaks of the C, O, and Mn elements and the weight percentage (Wt%) of each element. From Figure 2a–d, it is evident that the Mn, C, and O elements are evenly distributed across the surface of the carbon fibers, which proves that MnO_x_ has been successfully deposited on the surface of the carbon cloth with a good deposition effect. In order to evaluate the adsorption potential of the electrodes for heavy metal ions, the MnO_x_−1/CC electrodes were placed in ABS buffer pH = 5.5 containing 5 μM Cd^2+^, stirred thoroughly for 60 s without voltage applied, and then taken out and dried, and analyzed by EDS. As a result, as shown in Figure S1(a_1_–a_5_), the Cd, Mn, C, and O elements were uniformly distributed on evenly distributed on the surface of the carbon filaments, which strongly confirmed that the MnO_x_−1/CC electrodes possessed a good adsorption capacity for Cd^2+^ and were able to effectively capture heavy metal ions.

**Figure 3:**
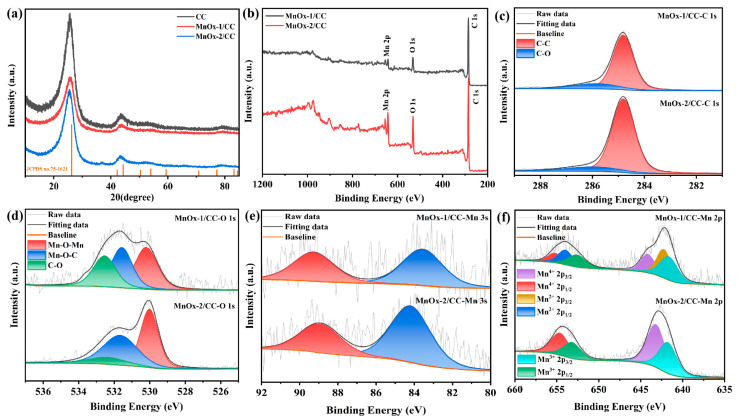
(**a**) XRD patterns of MnO_x_−1/CC and bare CC. (**b**) XPS spectra of MnO_x_−1/CC and MnO_x_−2/CC, (**c**) high-resolution XPS spectra of C 1s, (**d**) O 1s, (**e**) Mn 3s, and (**f**) Mn 2p.

Figure 3a indicates that the diffraction peaks of the MnO_x_−1/CC and MnO_x_−2/CC electrodes align with those of the original carbon cloth. This indicates that the introduction of manganese oxide did not significantly affect the crystal structure of the carbon cloth. The diffraction peaks at 2θ ≈ 26.2° and 44.3° correspond to the (002) and (101) crystal planes of the carbon material (JCPDS no. 75-1621). However, the strength of the MnO_x_−1/CC and MnO_x_−2/CC electrodes is significantly weaker compared with the bare carbon cloth. MnO_x_−1/CC did not appear to have obvious new diffraction peaks, and the diffraction peak of MnO_x_−2/CC at 37.12° is attributed to the (211) crystal face of MnO_2_ [35,36]. As shown in Figure 3b, the complete XPS spectra of MnO_x_−1/CC and MnO_x_−2/CC show the presence of Mn, O, and C elements, which proves the successful generation of MnO_x_ on the carbon cloth substrate. In Figure 3c, the C 1s spectra of the MnO_x_−1/CC and MnO_x_−2/CC samples are illustrated, which show the C 1s peaks at 284.8 eV and 285.84 eV. Two peaks, corresponding to the C-C and C-O bonds of the MnO_x_−1/CC sample, respectively, and the C-O bond binding energy of the MnO_x_−2/CC sample is slightly different at 285.94 eV compared with that of MnO_x_−1/CC. In the high-resolution XPS spectra at O 1 s (Figure 3d), the three peaks with binding energies of 530.2 eV, 531.57 eV, and 532.54 eV in the MnO_x_−1/CC sample corresponded to Mn-O-Mn, Mn-O-C, and C-O [37], respectively. The three peaks with binding energies of 530.02 eV, 531.73 eV, and 532.14 eV correspond to the Mn-O-Mn, Mn-O-C, and C-O bonds of the MnO_x_−2/CC sample, respectively. The presence of Mn-O-C bonds responds to the interaction between MnO_x_ and carbon cloth [37].

The multivalent transition of Mn is a key factor affecting the electron transfer efficiency of the electrode and its detection performance for heavy metal ions. As shown in Figure 3e, the Mn 3s peak emission spectra of the MnO_x_−1/CC and MnO_x_−2/CC electrodes indicate significant differences in the valence distributions of the two. The Mn 3s spin-orbital double-state energy level splitting (ΔE) of MnO_x_−1/CC is 5.71 eV, which is in between Mn^2+^ and Mn^3+^, which is consistent with the valence distribution shown in the high-resolution spectrum of Mn 2p in Figure 3f, suggesting that the Mn in the MnO_x_−1/CC samples exists mainly in the form of Mn^2+^ and Mn^3+^ [38,39], while the content of Mn^4+^ is low. The valence distributions shown in the high-resolution spectra of Mn 2p for the MnO_x_−1/CC sample are shown in Table 1. In contrast, the Mn 3s spin–orbit double-state energy level splitting (ΔE) of MnO_x_−2/CC is 4.72 eV, much closer to that of Mn^4+^ [40]. This result is consistent with the valence distribution shown in the high-resolution spectra of Mn 2p, as well as with the chemical species ratio data from the Mn 2p XPS spectra of the MnO_x_−2/CC samples in Table 1, which indicate that Mn^3+^ and Mn^4+^ are the dominant valence states of MnO_x_−2/CC, with Mn^4+^ being significantly higher. The difference in the valence distribution of Mn between the two electrode materials significantly affects their electrochemical performance in detecting Cd^2+^.

The above studies have shown that multivalent cycling of Mn plays a crucial role in Cd^2+^ detection. Due to the higher content of Mn^2+^ and Mn^3+^ in the MnO_x_−1/CC electrode and its stronger electron-supplying ability compared with Mn^4+^, the reduction of Cd^2+^ was enhanced, which led to the enrichment of more Cd^0^ on the electrode surface and the significant enhancement of electrochemical performance. In contrast, the dominance of Mn^4+^ in the MnO_x_−2/CC electrode, its strong oxidizing property, and lower electron supplying capacity limit the reduction of Cd^2+^, resulting in less enrichment of Cd^0^ on the electrode surface, which in turn leads to relatively poor electrochemical performance.

### 3.2. Electrochemical Properties

**Figure 4:**
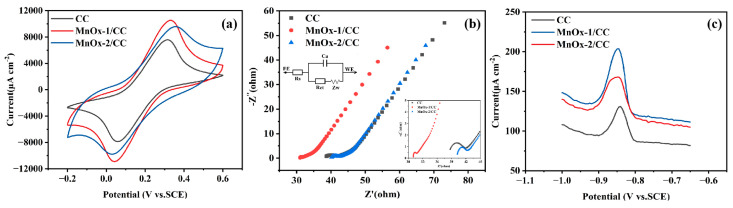
CV (**a**) and EIS (**b**) curves of MnO_x_−1/CC, MnO_x_−2/CC and CC in 5.0 mmol L^−1^ [Fe(CN)_6_]^3−/4−^ (+0.1 mol L^−1^ KCl) (CV scan rate: 100 mV s^−1^), and the DPV response for 5 μM Cd^2+^ in 0.1 mol L^−1^ ABS buffer (pH 5.5) (**c**).

**Figure 5:**
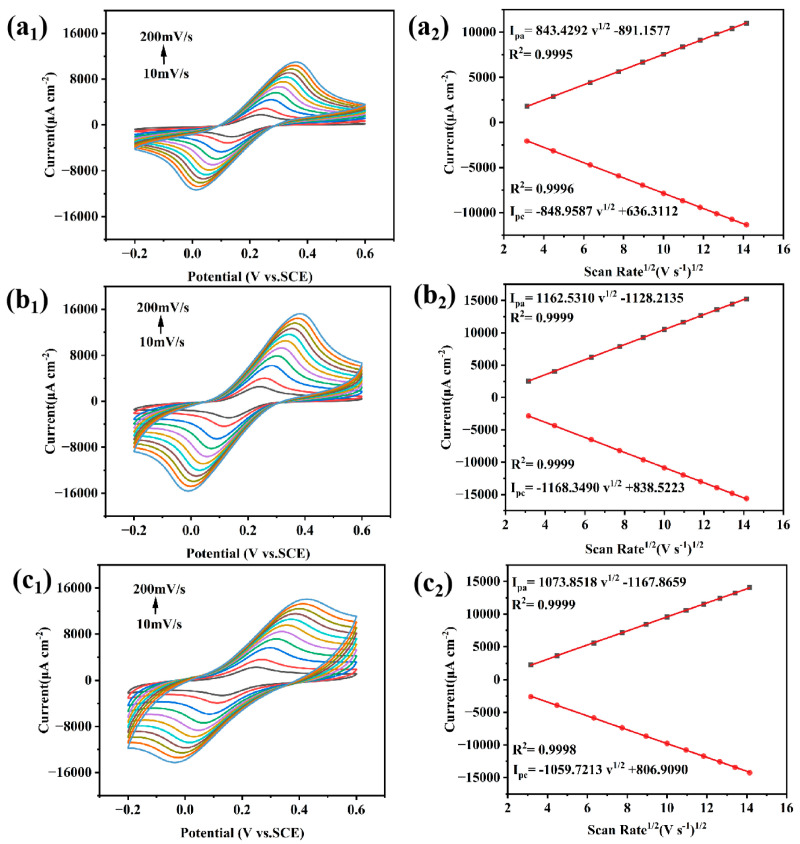
CVs of CC (**a_1_**,**a_2_**), MnO_x_−1/CC (**b_1_**,**b_2_**), and MnO_x_−2/CC (**c_1_**,**c_2_**) in 5.0 mmol L^−1^ [Fe(CN)_6_]^3−/4−^ (+0.1 mol L^−1^ KCl) at different scanning rates (10−200 mV s^−1^) and linear relationships between the oxidation peaks, reduction peaks, and the square root of scanning rate.

In this study, the electrochemical performance of the electrodes was probed using CV (scan rate: 100 mV s^−1^) in [Fe(CN)_6_]^3−/4−^ redox probe. As shown in Figure 4a, the MnO_x_−1/CC electrode exhibits a higher peak redox current compared with the MnO_x_−2/CC electrode and the CC electrode. This result is generally in agreement with the electrochemical impedance spectroscopy (EIS) results. Figure 5 shows the CV responses of MnO_x_−1/CC, MnO_x_−2/CC, and CC electrodes at different scan rates. The oxidation and reduction peak currents of [Fe(CN)_6_]^3−/4−^ measured at the electrodes exhibit a linear relationship with the square root of the scan rate. According to electrochemical principles, this clearly shows that the electrode reaction is mainly controlled by diffusion processes. Further, based on the Randles–Sevcik equation, the effective areas of the CC, MnO_x_−1/CC, and MnO_x_−2/CC electrodes obtained by cyclic voltammetry were 0.2439 cm^2^, 0.3356 cm^2^, and 0.3044 cm^2^, respectively. The Nyquist plots for each electrode (shown in Figure 4b) show that MnO_x_− 1/CC (Rct = 0.12728 Ω) has the smallest electron transfer resistance. The Rct of CC and MnO_x_−2/CC are 2.995 Ω and 1.581 Ω, respectively. This indicates that the best electron transfer efficiency is achieved at the interface of the MnO_x_−1/CC electrode.

To verify the feasibility of the MnO_x_/CC sensing electrode for the detection of Cd^2+^, we performed differential pulse voltammetry (DPV) experiments in 0.1 M acetate buffer solution (ABS). As shown in Figure 4c, the electrochemical responses of MnO_x_−1/CC, MnO_x_−2/CC, and bare CC electrodes were compared for the detection of 5 μM Cd^2+^, respectively. The results show that the peak current response of the MnO_x_−1/CC electrode was significantly higher than that of the MnO_x_−2/CC and bare CC electrodes, indicating that the MnO_x_−1/CC electrodes prepared by cyclic voltammetry exhibited a more superior Cd^2+^ detection electrochemical performance. This may be due to the larger effective active area of the MnO_x_−1/CC electrode, which provides more active sites and helps to increase the adsorption capacity for Cd^2+^. In addition, the higher content of Mn^2+^ and Mn^3+^ in the MnO_x_−1/CC electrodes and the strong electron-donating ability of these valence states promoted the reduction reaction of Cd^2+^, thus enhancing the adsorption and detection of Cd^2+^.

### 3.3. Electrochemical Detection of Cd^2+^

**Figure 6:**
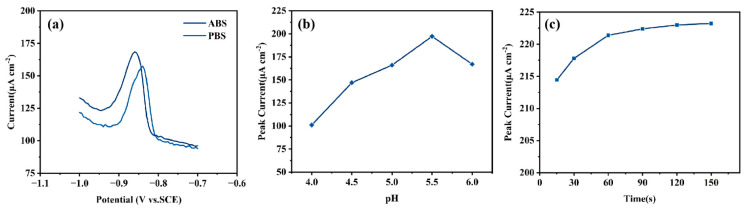
The effects of electrolyte (**a**), pH (**b**), and stirring time (**c**) on the DPV peak current of MnO_x_−1/CC in a 0.1 M buffer solution containing 5.0 μM Cd^2+^ are supported.

To optimize the experimental conditions for Cd^2+^ detection at the MnO_x_−1/CC electrode, we systematically investigated the effects of the choice of supporting electrolyte, the pH of the electrolyte, and the stirring time without pre-enrichment on the electrode’s detection performance. Figure 6a shows the dissolution voltammetric response of the MnO_x_−1/CC electrode for the detection of 5 μM Cd^2+^ in ABS buffer solution (0.1 M NaAc-HAc) and PBS buffer solution (0.1 M K_2_HPO_4_-KH_2_PO_4_). The results indicate that the peak current is significantly higher in the ABS buffer solution than in the PBS buffer solution; thus, the ABS buffer solution was chosen as the optimal supporting electrolyte. Subsequently, we optimized the pH of the ABS buffer solution. We examined the electrochemical signal response of the MnO_x_−1/CC electrode to 5 μM Cd^2+^ in the pH range of 4.0 to 6.0 (Figure 6b). The results show that the peak current of Cd^2+^ gradually increased with the rise in pH but reached a maximum at pH = 5.5 and then began to decrease. Therefore, the ABS buffer solution at pH = 5.5 was selected as the optimal supporting electrolyte for the subsequent experiments. To further optimize the effect of pre-enrichment-free stirring time on detection performance, we examined the response of the stirring time in the range of 15 s to 150 s. The response of the stirring time within this range is shown in Figure 6c. The experimental results indicate that the peak current gradually increased with the stirring time and stabilized after 60 s. This indicates that a stirring time of 60 s is sufficient to reach the adsorption equilibrium of Cd^2+^ on the electrode surface; thus, 60 s was adopted as the without pre-enrichment stirring time for the subsequent experiments.

**Figure 7:**
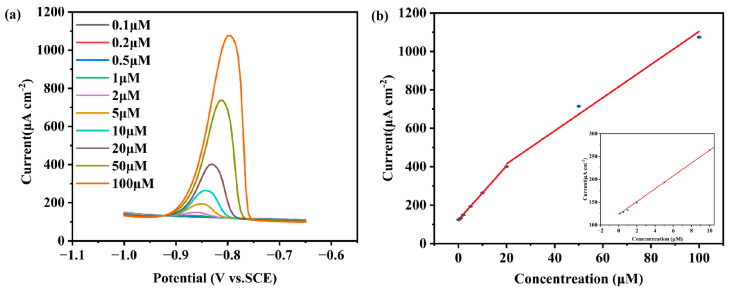
DPV response of MnO_x_−1/CC to different concentrations of Cd^2+^ (0.1−100.0 μM) (**a**) and calibration curve of Cd^2+^ (**b**).

Based on the above-optimized conditions, the electrochemical responses of different concentrations of Cd^2+^ were evaluated. As shown in Figure 7a, the peak currents were enhanced with the increase in Cd^2+^ concentration (0.1 μM to 100 μM), indicating that the electrodes demonstrated good response performance. Figure 7b shows the linear relationship between Cd^2+^ concentration and peak current, where the linear fitting equation in the range of 20 μM to 100 μM is I_p_ (μA) = 242.232 + 8.620C (μM) (R^2^ = 0.987). In the range of 0.1 μM to 20 μM, the linear fitting equation is I_p_ (μA) = 123.196 + 14.044C (μM) (R^2^ = 0.999). The limit of detection (LOD) of Cd^2+^ was calculated to be 0.08 μM (LOD = 3σ/S, signal standard deviation (σ), and slope of calibration curve (S) for blank samples). The experimental results show that the MnO_x_−1/CC electrode exhibited excellent detection sensitivity and linear response to Cd^2+^ under conditions of no pre-enrichment, demonstrating its potential for applications in the detection of heavy metal ions. It illustrates its applicability in the field of heavy metal ion detection.

Comparing the MnO_x_−1/CC electrode with electrodes also used for Cd^2+^ detection in recent years (e.g., Table 2), it was found that the MnO_x_−1/CC electrode did not show a significant advantage in terms of detection performance (e.g., detection range and detection limit). This difference mainly originated from the cancellation of the negative potential pre-enrichment step commonly used in dissolution voltammetry in this experiment. This resulted in a lower deposition of Cd^2+^ on the electrode surface than the pre-enrichment step. However, the advantage of detection without pre-enrichment is shorter detection time and higher efficiency. The performance of the electrode in heavy metal ion detection can be further improved in the future by further investigating how to improve the adsorption capacity of the MnO_x_ electrode for heavy metal ions.

### 3.4. Electrochemical Detection Strategies for Cd^2+^

Different valence states of Mn exist on the surface of the MnO_x_−1 electrode. These valence states create a specific charge distribution on the surface. As a cation, Cd^2+^ may be attracted to the charge on the manganese oxide surface, leading to adsorption. During this adsorption process, Cd^2+^ interacts electrostatically with Mn^2+^ and Mn^3+^ present on the surface of the manganese oxide. This interaction may also involve the transfer or redistribution of electrons.

**Figure 8:**
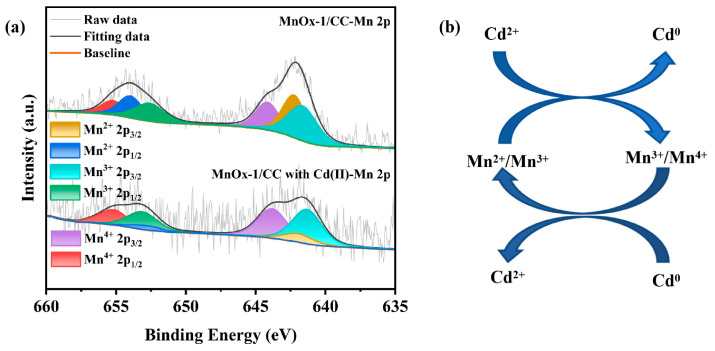
(**a**) High-resolution XPS spectra of Mn 2p before and after adsorption of 5 μM Cd^2+^ by MnO_x_−1/CC. (**b**) Cd(II) detection based on MnO_x_−1/CC adsorption and redox.

To further explore the mechanism of Mn’s role in the detection of Cd^2+^, Figure 8a analyses the changes in the surface electronic states of MnO_x_−1/CC during the adsorption of Cd^2+^. According to Table 3, the ratios of chemical species in the Mn 2p XPS spectra before and after adsorption of Cd^2+^ by MnO_x_−1/CC showed a significant decrease in the ratio of Mn^2+^ and an increase in the ratios of Mn^3+^ and Mn^4+^, which indicated that during the adsorption process, Mn^2+^ and Mn^3+^ were oxidized and involved in the Cd^2+^ reduction reaction, thus promoting the enrichment of Cd^0^ on the electrode surface. Notably, during the dissolution phase of dissolution voltammetry (DPV), the zero-valent metal (Cd^0^) deposited on the electrode surface was oxidized to cations (Cd^2+^), generating significant dissolution currents with peaks proportional to the concentration of Cd^2+^. In this process, the oxidative dissolution of Cd^0^ is further facilitated by the reduction reactions of Mn^3+^ and Mn^4+^. In the scheme of Figure 8b, the reaction mechanism for the detection of Cd^2+^ for MnO_x_−1/CC is as follows:

Mn(II) +Cd(II) → Mn(III) + Cd(0) (ads)

Mn(III) +Cd(II) → Mn(IV) + Cd(0) (ads)

Mn(IV) + Cd(0) →Mn(III) + Cd(II) (DPV)

Mn(III) + Cd(0) → Mn(II) + Cd(II) (DPV)

### 3.5. Selectivity, Repeatability, Reproducibility, Stability, and Real Sample Analysis

**Figure 9:**
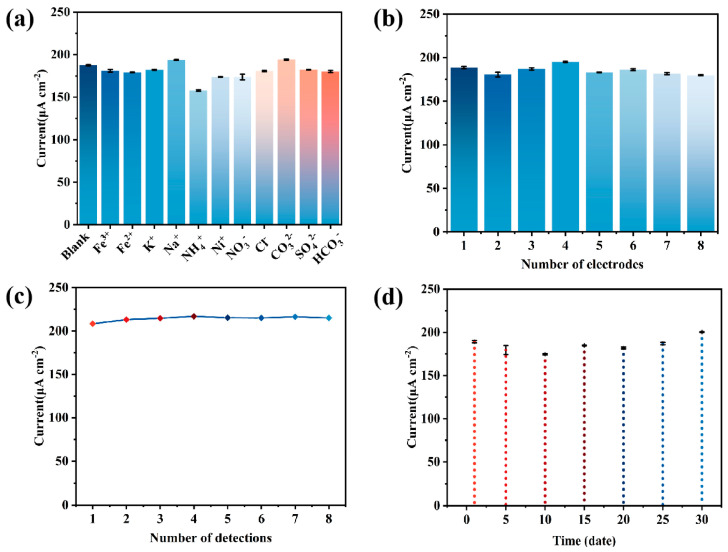
Interference test (**a**), repeatability (**b**), reproducibility (**c**), and stability test (**d**) of MnO_x_−1/CC against 5.0 μM Cd^2+^.

To evaluate the electrode materials’ anti-interference performance, a variety of interfering ions that may be present in the water samples were introduced into the experiment, including Fe^3+^, Fe^2+^, K^+^, Na^+^, NH_4_^+^, Ni^+^, Cl^−^, CO_3_^2−^, SO_4_^2−^, and HCO_3_^−^. The concentration of the interfering ions was 50 μM. The results show that the presence of these interfering ions had almost no significant effect on the detection performance of the electrode at 5 μM Cd^2+^, as shown in Figure 9a, indicating that the MnO_x_/CC electrode possesses good anti-interference capability. In addition, to verify the repeatability and reproducibility of the electrode, eight independent MnO_x_/CC electrodes were prepared using the same method for the detection of 5 μM Cd^2+^, resulting in a relative standard deviation (RSD) of 2.764%, as shown in Figure 9b. Meanwhile, eight parallel tests were performed on 5 μM Cd^2+^ using the same electrode, yielding an RSD of 1.239%, as shown in Figure 9c, further demonstrating the electrode’s excellent repeatability and reproducibility. The long-term stability of the electrode is crucial for practical applications. In this study, the MnO_x_/CC electrode was stored at room temperature for 1, 5, 10, 15, 20, 25, and 30 days and used for the detection of 5 μM Cd^2+^ under the same conditions. The results are shown in Figure 9d, and the RSD of the peak current was 4.412%, indicating that the electrode maintained good stability under long-term storage conditions. These results demonstrate that the MnO_x_/CC electrode exhibits excellent immunity to interference, reproducibility, and long-term stability, which lays the foundation for its application in the detection of heavy metal ions in real water samples.

To verify the ability of the MnO_x_/CC electrode to detect Cd^2+^ in real water samples, the performance of the electrode in tap water was systematically evaluated in this study using the standard addition method. The experimental results (e.g., Table 4) show that the recoveries of Cd^2+^ in tap water sample 1 were in the range of 102.0% to 104.2%, with relative standard deviations of less than 1.458%. The recoveries of Cd^2+^ in sample 2 were in the range of 103.2% to 110.0%, with the relative standard deviations less than 0.874%. This indicates that the electrode can be reliably applied to the determination of Cd^2+^ in water samples.

## 4. Conclusions

In this study, MnO_x_/CC electrodes were successfully prepared using a simple and efficient one-step electrodeposition method, and pre-enrichment-free detection of Cd^2+^ in water samples was achieved under optimized conditions. This research delves into the key effects of the valence composition and morphological characteristics of MnO_x_ electrode materials, which were constructed using various electrochemical methods, on the detection performance of Cd^2+^. The findings reveal that the valence cycle between Mn^2+^, Mn^3+^, and Mn^4+^ acts as an ’electron bridge’, significantly enhancing electron transfer efficiency and accelerating the redox reaction of Cd^2+^. Furthermore, an electrode morphology that boasts a larger effective active area and more active sites enhances the adsorption capacity for Cd^2+^. Future studies can further enhance the redox capacity and detection performance of Mn oxide electrodes by precisely modulating the valence ratio of Mn and nanostructure design. This study offers valuable design insights for the development of high-performance electrochemical sensors based on manganese oxide for heavy metal detection applications.

## Figures and Tables

**Table 1:** Proportions of chemical species in the Mn 2p XPS spectra of MnO_x_−1/CC and MnO_x_−2/CC.

Samples	Percentage of Manganese Species (%)		
	Mn^2+^	Mn^3+^	Mn^4+^
MnO_x_−1/CC	37.37	38.13	24.49
MnO_x_−2/CC	0	41.47	58.53

**Table 2:** Comparison of MnO_x_−1/CC electrode and other electrodes for Cd^2+^ detection.

Electrode	Enrichment Potential/V	Enrichment Time/s	Linear Range (mol/L)	LOD (mol/L)	Ref.
GC/GQDs-NF	−1.3	150	20~20 μg/L	11.3 μg/L	[13]
Au NPS/PT-N-RGO/SPE	−1.3	180	3~30 μg/L	0.46 μg/L	[14]
GC/rGO-SbNPs	−1.2	150	5 × 10^−8^~1.20 × 10^−7^	2.05 × 10^−8^	[41]
SiO_2_/GCE	−1	300	5 × 10^−7^~2.5 × 10^−6^	5 × 10^−7^	[42]
ZnO@SiO_2_/GCE	−1	300	2.5 × 10^−11^~1.75 × 10^−10^ 2.0 × 10^−10^~1.75 × 10^−9^	4.4 × 10^−11^	[42]
MnOx/CC	-	-	1 × 10^−7^~1 × 10^−4^	8 × 10^−8^	This work

**Table 3:** Proportions of chemical species in the Mn 2p XPS spectra of MnO_x_−1/CC after the initial measurement and adsorption of 5 μM Cd(II).

Samples	Percentage of Manganese Species (%)		
	Mn^2+^	Mn^3+^	Mn^4+^
Initial	37.37	38.13	24.49
After the adsorption of 5 μM Cd(II)	11.91	47.34	40.75

**Table 4:** Electrochemical detection of Cd^2+^ in tap water.

Actual Samples	Initial (μM)	Added (μM)	Found (μM)	Recovery (%)	RSD (%, *n* = 3)
Sample 1	0	1.0	1.02	102.0	1.458
		5.0	5.21	104.2	0.251
Sample 2	0	1.0	1.10	110.0	0.874
		5.0	5.16	103.2	0.285

## Data Availability

Data are contained within the article and Supplementary Materials.

## Supplementary Materials

The following supporting information can be downloaded at https://www.mdpi.com/article/10.3390/s25051415/s1, Figure S1: (a1) MnO_x_−1/CC (after Cd^2+^ adsorption), (a2) C, (a3) O, (a4) Mn,(a5) Cd. Figure S2: DPV response for electrochemical detection of Cd^2+^ in tap water (Sample 1). Figure S3: DPV response for electrochemical detection of Cd^2+^ in tap water (Sample 2).

## References

[1] Liu Q., Wang H., Ji J., Zhang W., Wang A., Zhao B., Chen Z. (2023). Cu-doped graphitic carbon nitride composite functionalized sensor for sensitive Cd^2+^ detection. Comput. Electron. Agric..

[2] Chu Y., Gao F., Gao F., Wang Q. (2019). Enhanced stripping voltammetric response of Hg^2+^, Cu^2+^, Pb^2+^ and Cd^2+^ by ZIF-8 and its electrochemical analytical application. J. Electroanal. Chem..

[3] Chen Y., Zhao P., Liang Y., Ma Y., Liu Y., Zhao J., Hou J., Hou C., Huo D. (2023). A sensitive electrochemical sensor based on 3D porous melamine-doped rGO/MXene composite aerogel for the detection of heavy metal ions in the environment. Talanta.

[4] Yu H., Zhao Q. (2024). Sensitive electrochemical sensor for Cd^2+^ with engineered short high-affinity aptamer undergoing large conformation change. Talanta.

[5] Chaudhary Y., Suman S., Rakesh B., Ojha G.P., Deshpande U., Pant B., Sankaran K.J. (2024). Boron and Nitrogen Co-Doped Porous Graphene Nanostructures for the Electrochemical Detection of Poisonous Heavy Metal Ions. Nanomaterials.

[6] Dong J., Wen L., Zhao D., Yang H., Zhao J., Hu Z., Ma Y., Hou C., Huo D. (2023). Flexible carbon fiber cloth supports decorated with cerium metal-organic frameworks and multi-walled carbon nanotubes for simultaneous on-site detection of Cd^2+^ and Pb^2+^ in food and water samples. Food Chem..

[7] GadelHak Y., Hafez S.H., Mohamed H.F., Abdel-Hady E., Mahmoud R. (2023). Nanomaterials-modified disposable electrodes and portable electrochemical systems for heavy metals detection in wastewater streams: A review. Microchem. J..

[8] Gao C., Huang X.J. (2013). Voltammetric determination of mercury (II). TrAC Trends Anal. Chem..

[9] Borrill A.J., Reily N.E., Macpherson J.V. (2019). Addressing the practicalities of anodic stripping voltammetry for heavy metal detection: A tutorial review. Analyst.

[10] Lu Y., Liang X., Niyungeko C., Zhou J., Xu J., Tian G. (2018). A review of the identification and detection of heavy metal ions in the environment by voltammetry. Talanta.

[11] Shi E., Yu G., Lin H., Liang C., Zhang T., Zhang F., Qu F. (2019). The incorporation of bismuth (III) into metal-organic frameworks for electrochemical detection of trace cadmium (II) and lead (II). Microchim. Acta.

[12] Tang L.W., Alias Y., Woi P.M. (2024). Simultaneous quantification of cadmium and lead ions using β-cyclodextrin functionalized poly (amino acid) electrochemical sensor. J. Electroanal. Chem..

[13] Pizarro J., Segura R., Tapia D., Navarro F., Fuenzalida F., Aguirre M.J. (2020). Inexpensive and green electrochemical sensor for the determination of Cd (II) and Pb (II) by square wave anodic stripping voltammetry in bivalve mollusks. Food Chem..

[14] Liang J., Zhang S., Huang Q., Li G., Zhou Z. (2024). Application of nitrogen-doped reduced graphene oxide-persimmon tannin nanocomposites for electrochemical detection of Cd (II) in water resources. J. Electrochem. Soc..

[15] Gao J., Yin J., Wang G., Wang X., Zhang J., Sun B., He D., Suo H., Zhao C. (2024). A novel electrode for simultaneous detection of multiple heavy metal ions without pre-enrichment in food samples. Food Chem..

[16] Gupte T., Jana S.K., Mohanty J.S., Srikrishnarka P., Mukherjee S., Ahuja T., Sudhakar C., Thomas T., Pradeep T. (2019). Highly sensitive As^3+^ detection using electrodeposited nanostructured MnO_x_ and phase evolution of the active material during sensing. ACS Appl. Mater. Interfaces.

[17] Xu H., Wang Q.Y., Jiang M., Li S.S. (2024). Application of valence-variable transition-metal-oxide-based nanomaterials in electrochemical analysis: A review. Anal. Chim. Acta.

[18] Li S.S., Xu Q.Q., Xu J.T., Yan G., Zhang Y.X., Li S.W., Yin L.C. (2022). Engineering Co^2+^/Co^3+^ redox activity of Ni-mediated porous Co_3_O_4_ nanosheets for superior Hg (II) electrochemical sensing: Insight into the effect of valence change cycle and oxygen vacancy on electroanalysis. Sens. Actuators B Chem..

[19] Liu Z., Xia X., Ye C.-J., Xu H., Wang Q.-Y., Zheng Z.-Y., Li S.-S., Liu Z., Guo Z. (2023). Sensitive sensing of Hg (II) based on lattice B and surface F co-doped CeO_2_: Synergies of catalysis and adsorption brought by doping site engineering. Anal. Chim. Acta.

[20] Gao Z.-W., Li H., Li P.-H., Li Y.-Y., Quan J.-Q., Ma N., Chen S.-H., Huang X.-J., Song Z.-Y., Yang M. (2024). In-situ precipitation zero-valent Co on Co_2_VO_4_ to activate oxygen vacancies and enhance bimetallic ions redox for efficient detection toward Hg (II). Anal. Chim. Acta.

[21] Wang X.-Q., Ma X.-Y., Wu W.-Z., He H.-B., Wang N.-N., Zheng R.-J., Ma S.-J., Zhu Y.-Q., Shen P.-K., Zhu J.-L. (2024). MnS–MnO heterogeneous nanocube@ N, S-doped carbon as a highly efficient bifunctional water splitting electrocatalyst. Rare Met..

[22] Zhang Y., Gao Y., Wang Q. (2024). Enhanced catalytic oxidation of toluene with a MnO_x_/copper mesh monolithic catalyst prepared using one-step electrodeposition method. Mol. Catal..

[23] Pan G., Hu Y., Wang Z., Li H., Wu D., Zhang L., Zhang J. (2024). Metal–Organic Framework-Derived MnO/C Nanocomposite with Lamellar Porous Structure for High-Performance Aqueous Zinc-Ion Battery. Energy Technol..

[24] Sun Y., Ba Y., Sun J. (2024). S-MnO_2_/Graphene composites with high electrochemical properties as the cathode of aqueous zinc-ion battery. Mater. Lett..

[25] Zhang S., Wang Z., Yang S., Hao D., Yu S., Wu Q. (2024). Chitosan modified graphene oxide with MnO_2_ deposition for high energy density flexible supercapacitors. Int. J. Biol. Macromol..

[26] Moyseowicz A., Kordek-Khalil K., Moyseowicz A. (2024). Controlled preparation of carbon cloth decorated with nanostructured Mn (OH)_2_/Mn_3_O_4_ electrodes for high-performance asymmetric supercapacitors. Chem. Eng. Process.-Process Intensif..

[27] Singh A.K., Keshari P., Saroj A., Ramanathan V. (2023). Electrodeposition of Graphitic Carbon Nitride and its In situ Decoration with MnO_2_ Nanostructures: A Tailored Interface for Dopamine Sensing. Surf. Interfaces.

[28] Liu R., Zhang C.J., Han X., Wu T.H., Liu R.J., Sun Y., Jin S. (2023). MnO_2_/graphene supported on Ni foam: An advanced electrode for electrochemical detection of Pb (II). Carbon Lett..

[29] Liu F., Geng L., Ye F., Zhao S. (2022). MOF-derivated MnO@C nanocomposite with bidirectional electrocatalytic ability as signal amplification for dual-signal electrochemical sensing of cancer biomarker. Talanta.

[30] Peera S.G., Koutavarapu R., Reddy P.S.P., Koyyada G., Alodhayb A.N., Pandiaraj S., Kim S.W., Tamtam M.R. (2024). Effect of N-doped carbon on the morphology and oxygen reduction reaction (ORR) activity of a xerogel-derived Mn (II) O electrocatalyst. Catalysts.

[31] Du X., Hou C., Kimura H., Song J., Yang X., Xie X., Jiang H., Zhang X., Sun X., Zhang Y. (2024). Restricted and epitaxial growth of MnO_2-x_ nano-flowers in/out carbon nanofibers for long-term cycling stability supercapacitor electrodes. J. Colloid Interface Sci..

[32] Chen L., Gan Q., Xiao X., Cai S., Yan X., Zheng C. (2024). Bio-templated synthesis of MnO_2_-based micromotors for enhanced heavy metal removal from aqueous solutions. J. Mater. Sci..

[33] Kim E.-J., Lee C.-S., Chang Y.-Y. (2013). Hierarchically structured manganese oxide-coated magnetic nanocomposites for the efficient removal of heavy metal ions from aqueous systems. ACS Appl. Mater. Interfaces.

[34] Li W., Li Y., Liu J., Chao S., Yang T., Li L., Wang C., Li X. (2021). A novel hollow carbon@ MnO_2_ electrospun nanofiber adsorbent for efficient removal of Pb^2+^ in wastewater. Chem. Res. Chin. Univ..

[35] Rajagopal R., Ryu K.S. (2018). Influence of rare earth elements on porosity controlled synthesis of MnO_2_ nanostructures for supercapacitor applications. Electrochim. Acta.

[36] Zhou J., Qin L., Xiao W., Zeng C., Li N., Lv T., Zhu H. (2017). Oriented growth of layered-MnO_2_ nanosheets over α-MnO_2_ nanotubes for enhanced room-temperature HCHO oxidation. Appl. Catal. B Environ..

[37] Lin R., Xiao M., Xu Y., Zeng L., Zhu F., Zhang Y., Meng Y. (2024). MnO_2_ nanoflakes anchored on carbon nanotubes as self-standing anode for sodium ion batteries. J. Energy Storage.

[38] Zhao W.K., Sun B.C., Han C.B., Zhou K.L., Wang C., Zheng J.Y., Lu Y., Fang D., Yan H. (2023). Anion assisted completely reconfigured manganese oxides with optimal proton adsorption for boosting acidic hydrogen evolution reaction. Chem. Eng. J..

[39] Ji F., Men Y., Wang J., Sun Y., Wang Z., Zhao B., Tao X., Xu G. (2019). Promoting diesel soot combustion efficiency by tailoring the shapes and crystal facets of nanoscale Mn_3_O_4_. Appl. Catal. B Environ..

[40] Nagaraju G., Ko Y.H., Cha S.M., Im S.H., Yu J.S. (2016). A facile one-step approach to hierarchically assembled core–shell-like MnO_2_@MnO_2_ nanoarchitectures on carbon fibers: An efficient and flexible electrode material to enhance energy storage. Nano Res..

[41] Nunes E.W., Silva MK L., Cesarino I. (2020). Evaluation of a reduced graphene oxide-Sb nanoparticles electrochemical sensor for the detection of cadmium and lead in chamomile tea. Chemosensors.

[42] Dhaffouli A., Holzinger M., Carinelli S., Barhoumi H., Salazar-Carballo P.A. (2024). ZnO Doped Silica Nanoparticles (ZnO@ SiO_2_) for Enhanced Electrochemical Detection of Cd^2+^ Ions in Real Samples. Sensors.

