# Supplementary material for: Preparation of MnOx/CC Electrode by One-Step Electrodeposition for Electrochemical Detection of Cd2+ in Water
Source: Sensors (Basel). 2025 Feb 26;25(5):1415. doi: 10.3390/s25051415 (PMC11902686; doi:10.3390/s25051415)
Supplement: Supplementary file 1 [file sensors-25-01415-s001.zip › sensors-3460628-supplementary.pdf]

Supplementary Materials

Preparation of MnO_x/CC electrode by one-step electrodeposition for the electrochemical detection of Cd^{2+} in water

Jun Yin [1], Haiyang Huang [1], Cong Zhao [2], Haoyu Zhu [1], Hui Suo [1], Dong He [1] and Chun Zhao [1,*]

Table of Contents

1. Figure

Figure S1. (a1) MnO_x−1/CC (after Cd^{2+} adsorption), (a2) C, (a3) O, (a4) Mn,(a5) Cd.
Figure S2. DPV response for electrochemical detection of Cd^{2+} in tap water (Sample 1).
Figure S3. DPV response for electrochemical detection of Cd^{2+} in tap water (Sample 2).

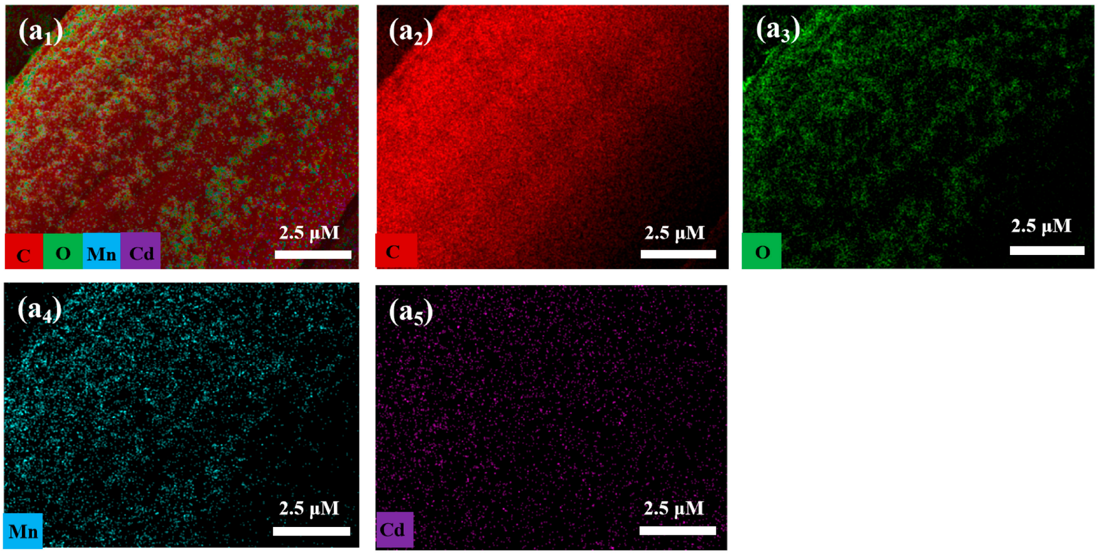

Figure S1. (a_1) MnO_x−1/CC (after Cd^{2+} adsorption), (a_2) C, (a_3) O, (a_4) Mn,(a_5) Cd.

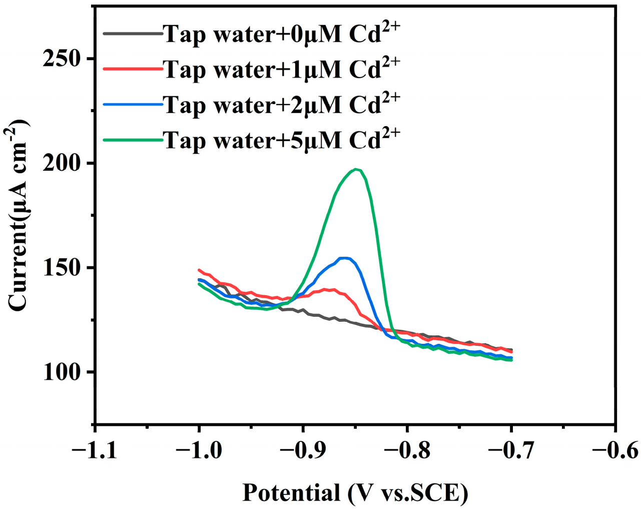

Figure S2. DPV response for electrochemical detection of Cd^{2+} in tap water (Sample 1).

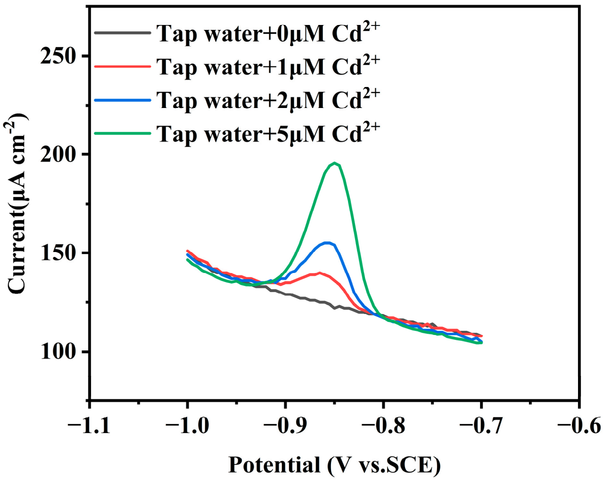

Figure S3. DPV response for electrochemical detection of Cd^{2+} in tap water (Sample 2).
